# Physical Cues of Matrices Reeducate Nerve Cells

**DOI:** 10.3389/fcell.2021.731170

**Published:** 2021-09-27

**Authors:** Yiqian Luo, Jie Li, Baoqin Li, Yuanliang Xia, Hengyi Wang, Changfeng Fu

**Affiliations:** Department of Spine Surgery, The First Hospital of Jilin University, Changchun, China

**Keywords:** physical cue, mechanical transduction, neurite, neural stem cell, nerve regeneration

## Abstract

The behavior of nerve cells plays a crucial role in nerve regeneration. The mechanical, topographical, and electrical microenvironment surrounding nerve cells can activate cellular signaling pathways of mechanical transduction to affect the behavior of nerve cells. Recently, biological scaffolds with various physical properties have been developed as extracellular matrix to regulate the behavior conversion of nerve cell, such as neuronal neurite growth and directional differentiation of neural stem cells, providing a robust driving force for nerve regeneration. This review mainly focused on the biological basis of nerve cells in mechanical transduction. In addition, we also highlighted the effect of the physical cues, including stiffness, mechanical tension, two-dimensional terrain, and electrical conductivity, on neurite outgrowth and differentiation of neural stem cells and predicted their potential application in clinical nerve tissue engineering.

## Role of Physical Cues in the Regulation of Nerve Cell Behavior

Currently, clinical treatment strategies for nerve injury are varied, including autotransplantation, decompression surgery, hormone shock, and life support, while the recovery of nerve function is unsatisfactory. After nerve injury, primary and secondary injuries cause a large number of cell deaths in the nervous system (Ahuja et al., 2017b). As the main undertaker of functional activities of the nervous system, neurons are non-renewable cells and cannot produce offspring by self-division to replace dead cells (Myers et al., 1981). With advances in neuroscience, researchers have found that neural stem cells (NSCs) in the adult mammalian central nervous system hold the key to neural regeneration through proper migration, differentiation, and maturation, to replace dead neurons and establish nascent neural networks, which can be integrated into damaged neural circuits to repair function (Yang Z. et al., 2015). However, primary and secondary injuries also destroy the extracellular matrix (ECM) and impede survival, migration, and differentiation of potential replacement cells of nerve cells in the nervous system, which is an important cause of nerve repair failure (Okada, 2016; Bradbury and Burnside, 2019). Recently, the regulation of nerve cell behavior by ECM remodeling has been considered the most promising strategy and has been widely explored in nerve injury repair. More importantly, reeducation of nerve cells, such as promoting robust growth of neurites and large-scale directional differentiation of NSCs into neurons by physical cues in the ECM, is a feasible method for the formation of new functional connections to potentiate repair of nerve injury (Yang Z. et al., 2015; Rao et al., 2018).

The regulation of nerve cell behavior by biochemical cues based on bioactive substances is often limited by their disadvantages, such as short half-life, easy inactivation, and low efficacy *in vivo* (Fornaro et al., 2020). Although various delivery systems based on biomaterials have been developed for the sustained release of bioactive substances with functional behavioral regulation, the effect is still not ideal because of the non-matched release profiles with nerve injury repair. With the development of neuroscience, it has been proven that nerve cells have a biological basis for performing mechanical transduction, consisting of integrins, mechanically sensitive ion channels, G proteins, second messengers, and the cytoskeleton, which can transform physical cues in the ECM, such as mechanical, topographic, and electrical signals, into intracellular biological signals to alter the behavior of nerve cells (Martinac, 2004). In contrast to bioactive substances, the physical cues in matrices tend to be relatively persistent and stable. In view of this, physical cues such as advantageous stiffness, topography, mechanical tension, and conductivity are being considered for integration into the preparation of neural tissue engineering scaffolds to regulate the behavior of nerve cells directionally, contributing to the repair of nerve injury (Liu et al., 2018; Amani et al., 2019).

**FIGURE 1:**
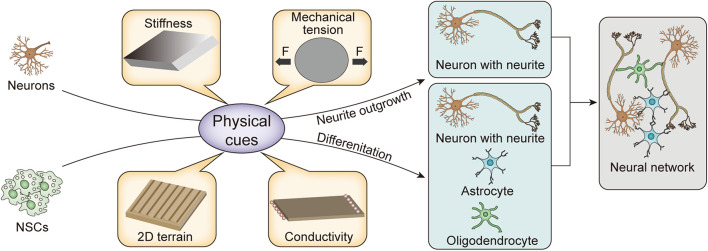
Schematic illustration of physical cues for remodeling nerve cells.

To systematically and comprehensively show the development of the physical cues and explain their mechanism, we review the biological basis and pathways of mechanical transduction from the perspective of molecular biology. In addition, we discuss the remodeling of neurite outgrowth and NSC differentiation through stiffness, mechanical tension, two-dimensional (2D) terrain, and conductivity provided by biomaterial matrices, as shown in Figure 1 and Table 1. Finally, the potential applications of physical cues in nerve tissue engineering are also discussed.

**TABLE 1:** Behavior regulation of neural cells by physical cures of biomaterial matrices.

Property	Biomaterial	Cell type	Result	References
Stiffness	HA	DRGs	The neural differentiation of NSCs has an optimal range. Outside this range, the ability to differentiate into neurons has decreased. The differentiation of glial cells is stronger on the hard matrix. There are also optimal ranges for neurite. In soft matrices, there are relatively few neurite and shorter lengths. In hard matrices, neurons have longer processes and more branches.	Khoshakhlagh and Moore (2015)
	PDMS, laminin	Hippocampal neurons		Wen et al. (2018)
	Polyvinyl alcohol	Bone marrow stem cells		Kim et al. (2015)
	Laminin-polylysine	Primary cortical neurons		Lantoine et al. (2016)
	PDMS	NSCs		Blaschke et al. (2019)
	Oligonucleotide-crosslinked ECM platform	NSCs		Rammensee et al. (2017)
	PEG	NSCs		Mosley et al. (2017)
Mechanical tension	Carbon nanotube, quartz	Invertebrate neurons	Stretching increases the length of major neurite but also reduces the number of protrusions per neuron. The direction of neurite growth is parallel to some extent to the direction of application of tension.	Anava et al. (2009)
	Laminin, fibronectin	NSPCs		Arulmoli et al. (2015)
	PLGA	E15 spinal motor neurons		Feng et al. (2016)
	PDMS	NSCs		Chang et al. (2013)
2D terrain	PCL	PC12 cells	Larger fiber diameters are more conducive to neural differentiation. In addition, the orientation of the nanofibers allows the neurite to run parallel to the nanofibers.	Panahi-Joo et al. (2016)
	PLGA	hNSCs		Yang K. et al. (2015)
	Fibrin	DRG		Yao et al. (2016)
	PLA	Schwann cells, motor neurons, DRG		Wang et al. (2015)
	PVDF	NSCs		Lins et al. (2017)
	PCL, PLA	Schwann cells		Mobasseri et al. (2015)
	PCL	NSCs		Czeisler et al. (2016)
Electrical conductivity	PPy, DBS	hNSCs	Electrical conductivity can promote the differentiation of NSCs into neurons and glial cells and can also inhibit the differentiation into glial cells. It can also promote the growth of neurite.	Stewart et al. (2015)
	TA, peptide amphiphile	PC12 cells		Arioz et al. (2018)
	Silver nanowire, PEG	NSCs		Lee et al. (2018)
	IrO_2_, Ir	NSCs, PC12 cells		Chen et al. (2019)
	PEDOT, HA, Cs, gelatin	NSCs		Wang et al. (2017)
	PANI, PS	NSCs		Xu et al. (2016)
	Carbon nanotube, graphene, Cs	Hippocampal neurons		Gupta et al. (2019)
	PPy, PLGA, PCL	DRG		Nguyen et al. (2014)
	Carbon nanotube, PCL	PC12 cells		Zhou Z. et al. (2018)

*DRG, dorsal root ganglia; hNSCs, human neural stem cells; NSPCs, neural stem progenitor cells; TA, tetraaniline; IrO_2_, iridium oxide; PS, polystyrene; DBS, dodecylbenzenesulfonate; HA, hyaluronic acid; PDMS, polydimethylsiloxane; PU, polyurethane; PES, polyethersulfone; PVDF, poly(vinylidene fluoride).*

## Biological Basis of Nerve Cell Mechanical Transduction

Nerve cells with mechanical sensors can sense changes in the external mechanical, topographical, and electrical microenvironment and transform physical cues into a series of biochemical signals inside the cell, ultimately leading to changes in gene expression (Martinac, 2001). During mechanical transduction, the ECM, cell membrane, cytoskeleton, and nucleus are connected to form a perfect tension integration system for operation (Provenzano and Keely, 2011; Chighizola et al., 2019).

The nerve cell membrane plays an important role during mechanical perception as a unit in direct contact with the outside world. The cell membrane is an elastic structure composed of a lipid bilayer and proteins. Under the action of an external force, the area of the cell membrane increases, the thickness is reduced, and the local curvature changes, simultaneously leading to the deformation of the lipid bilayer (Fletcher and Mullins, 2010). In addition, as the lipid bilayer changes, the conformation and activity of membrane proteins change, which ultimately leads to the activation of various signal transduction pathways. In particular, integrins, mechanically sensitive ion channel proteins, and G proteins are the most important membrane proteins during mechanical transduction and have attracted extensive attention (Hughes-Fulford, 2004; White and Frangos, 2007; Groves and Kuriyan, 2010). In addition, second messengers and the cytoskeleton in the cytoplasm also play an important role in mechanical signal transduction (Janmey and McCulloch, 2007).

### Integrins

Integrins are a family of membrane surface protein receptors, which are composed of two subunits, α and β. Both α and β subunits are single transmembrane glycoproteins, which are composed of the extracellular, intracellular, and transmembrane regions. Currently, more than 20 integrins have been identified, all of which are heterodimeric transmembrane glycoproteins (Cabahug-Zuckerman et al., 2018). In the process of integrin-mediated extracellular mechanical stimulus conversion into intracellular biochemical signals, the interaction between an integrin and the ECM is first required; second, the integrin and its ligand need to form a new connection. The extracellular membrane domain of an integrin is a large N-terminal domain that recognizes polypeptide sites containing arginine–glycine–aspartic acid sequences and binds to specific ligands (e.g., collagen, fibronectin, and laminin) in the ECM through changes in its folding and internal rotation (Humphries et al., 2019). The intracellular domain of integrins can bind to structural proteins, such as the cytoskeleton. In addition, it can bind to various signaling proteins, such as focal adhesion protein tyrosine kinases (FAK) and protein kinase C (PKC), which play an important role in signal transduction in both cellular and extracellular domains (Sun et al., 2016; Moreno-Layseca et al., 2019).

Once an integrin binds to a ligand in the ECM, integrin aggregation and protein kinase aggregation occur. The protein kinases and adaptor proteins move together to the tail inside the cytoplasm of the integrin β subunit and promote the connection to the actin cytoskeleton, leading to the formation of myosin tension fibers (Baaske et al., 2019). The rearrangement of the actin cytoskeleton further leads to the aggregation of other intracellular signaling factors, resulting in the formation of a complex known as the focal adhesion complex (FAC). The aggregation of signaling and cytoskeletal proteins causes various enzymes to contact their substrates, thereby triggering intracellular signal transduction (Servin-Vences et al., 2018). FAK, a calcium-independent tyrosine kinase belonging to the Src family, is one of the most important molecules for conjugation with the newly formed FAC. FAK is activated by most integrins, and once activated, autophosphorylation occurs, producing an SH2-binding site for Src or Fyn. Src kinase then phosphorylates various components clustered at the FAC, such as pile-tensin and P130Cas, leading to further signal transduction (Bachmann et al., 2019). On the one hand, Grb2/SOS enters the Ras pathway and activates mitogen-activated protein kinase (MAPK). On the other hand, FAK recombines with Src to activate MAPK by phosphorylating Cas and statin, which in turn connects to the guanosine triphosphate (GTP) exchange factor through the connector protein and enters the Ras pathway (Geoghegan et al., 2019; Orre et al., 2019).

### Mechanosensitive Ion Channels

**FIGURE 2:**
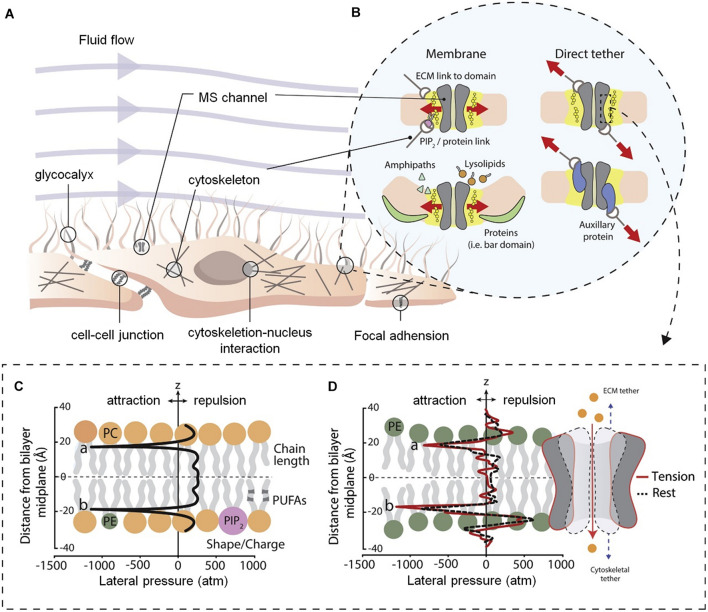
Diagram of cell membrane and gating models for mechanosensitive ion channels (Cox et al., 2019). **(A)** Structure of mammalian cell environment. **(B)** Bilayer and direct tether models of mechanosensitive (MS) channel gating. **(C)** Ideal pressure curves for symmetric bilayers. The profile can be altered by the properties of the embedded membrane proteins and constituent lipid components. **(D)** Calculated the pressure distribution of 1-palmitoyl-2-oleoyl-sn-glycero-3-phosphoethanolamine (POPE) double-layer molecular dynamics simulation at rest and apply tension. Figures reproduced with permission from the references listed.

In the body, a variety of cells, including nerve cells, can activate the cell signal transduction pathway through mechanically sensitive ion channels after sensing mechanical stimulation in the extracellular environment, affecting cell proliferation, differentiation, migration, and apoptosis (Figure 2A). The mechanically sensitive gated channels have two structures of double layer and direct tether. Both can be coincident, where the structural scaffold protein focuses the force into the mechanically sensitive channel domain, but the final transducer is a bilayer structure (Figure 2B). It is worth noting that pressure distribution across the pure bimolecular layer is symmetric. In each monolayer, there are negative pressure peaks caused by the surface tension of the water lipid interface and repulsive positive pressure peaks at the lipid head group and hydrophobic end (Figures 2C,D; Cox et al., 2019).

Eukaryotic mechanically sensitive channel proteins mainly include degenerin/epithelial sodium channels, transient receptor potential channels, two-pore-domain potassium channels, and Piezo proteins. In particular, Piezo, a newly discovered mechanically sensitive channel protein, plays a crucial role in the mechanical transduction process. Piezo was first identified by Coste et al. (2010). in a mouse neuroblastoma. Subsequently, a series of studies have confirmed that Piezo protein is widely expressed in the kidney, bladder, colon, blood vessel, lung, ganglion, and other tissues (Coste et al., 2010). They can non-selectively pass divalent ions and monovalent basic ions, which are important for the generation of mechanically gated non-selective cation currents. Piezo proteins constitute a family with two genetically similar proteins, Piezo1 and Piezo2, which are composed of approximately 2,500 amino acids and contain 24 to 36 transmembrane regions (Anava et al., 2009). These proteins are known to have the largest number of transmembrane regions and are not homologous to other mechanically sensitive or voltage-sensitive channel proteins currently known.

In particular, the Piezo2 protein is primarily present in neurons (Ingber, 2008). In the human body, the Piezo2 protein channel is mainly distributed in trigeminal sensory cells, dorsal root ganglion cells, Merkel cells, and somatic neuron cells (Martinac, 2004; Tay and Di Carlo, 2017). Studies on somatosensory neurons in mice have shown that the use of a selective exchange protein directly activated by a cyclic adenosine monophosphate (cAMP) 1 agonist enhanced Piezo2 expression, resulting in increased nerve sensitivity and a decreased pain threshold. After the Piezo2-related genes were knocked out, the nerve cells lost their ability to respond to the stimulus. When the Piezo2b gene fragment was specifically knocked out during the embryo stage, zebrafish lost almost all light tactile responses, suggesting that Piezo2 mediates both the pain and the tactile response to the mechanical stimulus (Llobet et al., 2018). Moreover, after blocking the expression of Piezo2 *via* small interfering RNA interference, dorsal root ganglion cells lost their ability to respond to the fast stimulus, suggesting that the expression of Piezo2 controls the generation of the fast adaptive current mediated by the mechanical stimulus.

### G Proteins

G proteins, also known as GTP-binding proteins, or guanine nucleoside regulatory proteins, are a special family of regulatory proteins. They are coupled to many membrane receptors and effectors in a specific way, which play an important role in the process of cell signal transmembrane transduction (Zhang et al., 2008). When the α subunit of the G protein is bound to guanosine diphosphate, the protein is inactive. Guanosine diphosphate dissociates as soon as the ligand binds to the corresponding receptor, resulting in changes in G protein configuration (Wang J. et al., 2018). GTP can then bind to the empty guanosine binding site to form α-GTP. With the participation of the magnesium ion, the α subunit is activated, and the G protein is decomposed into active α-GTP and βγ dimers to perform the task. Subsequently, the α subunit acts as a GTP enzyme and hydrolyzes the GTP attached to it into guanosine diphosphate. Then, α-GDP reassociates with βγ to restore the heterotrimeric inactive G-GDP complex, thus completing the cycle.

In recent years, many studies have suggested that large and small G proteins are important links in the mechanical stimulation of cells (Hong et al., 2016; Balemans et al., 2017). In particular, large G proteins are anchored to the cell membrane by lipid modification of amino acid residues on their subunits, thus providing a structural basis for them to receive structural signals from the cell membrane. Small G proteins, including Ras, Rab, Arf, Rho, and Sar, receive extracellular mechanical signals mainly through the ECM–integrin–cytoskeleton system (Wang J. et al., 2018; Cortes et al., 2019). In addition, Rab, Arf, and Sar are also involved in intracellular protein and vesicle transport; Ras plays a key role in mediating the MAPK phosphorylation cascade; and the Rho subfamily regulates cytoskeletal function, such as the ectopic recombination of cytoskeletal depolymerization to create new forms.

### Second Messengers

After sensing mechanical stress, nerve cells generate a series of second messenger molecules, such as calcium ions (Ca^2+^), cAMP, PKC, and inositol triphosphate. In particular, calcium ions constitute the most important second messenger and the most widely distributed signal molecule in cells. All types of extracellular signals can be transmitted to cells *via* Ca^2+^, causing a cascade of intracellular signals and regulating biological processes such as cell proliferation, differentiation, and secretion. For example, the regulation of genes related to cell proliferation and functional activation, such as *c-los*, *c-an*, and *c-myc*, requires the participation of Ca^2+^. Intracellular calcium occurs in two forms: binding calcium and free calcium (Zhang et al., 2020). In general, more than 99.9% of intracellular calcium is in the form of binding calcium, which is mainly distributed in the nucleus, mitochondria, endoplasmic reticulum, and plasma membrane, while intracellular free calcium is very rare.

The regulation of intracellular free Ca^2+^ concentration is a key factor in the process of information transfer. The concentration of cytoplasmic Ca^2+^ can be significantly increased when cells are stimulated by physical and chemical factors (Provenzano and Keely, 2011; Ranade et al., 2015). Changes in Ca^2+^ concentration are mainly realized through calcium channels on the membrane, including voltage-controlled calcium channels, mechanically controlled calcium channels, and receptor-controlled calcium channels. Voltage-controlled calcium channels are of great concern (Clausen et al., 2017). This pathway can be opened when the nerve cell is stimulated, and the membrane depolarizes. Channels are classified into three types according to their sensitivity to membrane voltages: L-type, T-type, and N-type. In particular, the L-type, with a large conductance, high threshold, and long opening time, can be blocked by traditional calcium channel blockers. Mechanically controlled calcium channels, also known as stretch-sensitive calcium channels, can directly induce mechanical signals to regulate Ca^2+^ signal conduction (Stukel and Willits, 2016). Campbell et al. (2008) added an L-type Ca^2+^ channel blocker to partially inhibit the biological effects of osteoblasts induced by mechanical stimulation. Peake et al. (2000) reported that the addition of extracellular Ca^2+^ receptors or mechanically sensitive calcium channel blockers could partially block the downstream activation of Ca^2+^. Therefore, the variation in intracellular Ca^2+^ concentration is considered an early response to mechanical stress and an important signal for nerve cell proliferation and differentiation.

### Cytoskeleton

The cytoskeleton consists mainly of microtubules, microfilaments, and intermediate fibers, which are networks of protein fibers inside the cell membrane (Blanquie and Bradke, 2018). Microtubules are hollow, cylindrical organelles with polarity and consist of tubulin and microtubule-binding proteins. In particular, tubulin has obvious homology with G proteins and has their functional characteristics (Kast and Dominguez, 2017). Microfilaments are helical structures composed of actin subunits. Actin exists in two forms, namely, active polymerized fibrous actin and inactive soluble globular actin, and there is a dynamic balance between the contents of these two forms. Intermediate fibers are fibrous proteins with diameters between microfilaments and microtubules, which can extend from the nuclear fiber layer through the cytoplasm and form a lattice structure in the cytoplasm (Halpern et al., 2017; Marsico et al., 2018). Although the three fiber components differ in morphological structure and function, they are interconnected to form a complex skeleton system that runs through the whole nerve cell and determines the terrain and rigidity of the nerve cell. In addition, the cytoskeleton is related to growth, differentiation, migration, apoptosis, intracellular information transfer, gene expression, and other important life activities (Provenzano and Keely, 2011; Konietzny et al., 2017; Svitkina, 2018).

The cytoskeleton, as the key of the whole signaling network, conducts mechanical signaling through physical and chemical pathways. These pathways coordinate with each other to complete the transfer and transformation of external mechanical stimuli. First, external forces can cause tension redistribution within the cytoskeleton, leading to the rearrangement of cytoskeletal fiber bundles, which mainly play a physical conduction role (Fletcher and Mullins, 2010; Provenzano and Keely, 2011). Mechanical stimulation of nerve cell membranes with different intensities immediately causes intracytoplasmic organelle translocation and nuclear deformation, even though the elasticity coefficient of the nucleus is almost 10 times that of the cytoplasm. When cytoskeletal protein depolymerizing agents (e.g., cytochalasin and acrylamide) are applied to destroy the microfilaments and intermediate filaments, nuclear stiffness is significantly reduced, suggesting that cytoskeleton proteins have a direct effect on nuclear structure (Lin et al., 2009).

Second, as a converter between mechanical and chemical signals, the adaptive changes in the cytoskeleton can increase the permeability of ion channels and the activity of some receptors and finally transmit mechanical signals to the nucleus with the help of a second messenger, regulating the expression of related genes (Mui et al., 2016; Hohmann and Dehghani, 2019). In terms of the integrin–cytoskeleton pathway, they can regulate the receptor tyrosine kinase/Ras/MAPK pathway by various methods such as regulating receptor tyrosine kinase activation, blocking Ras to root abundant factor signaling, and blocking extracellular signal-regulated kinase transfer to the nucleus. In addition, actin interacts with cadherin and α-catenin, leading to the release of β-catenin from the nerve cell membrane and binding to the transcription factor in the cytoplasm, both of which enter the human nucleus. In these ways, the cytoskeleton is involved in regulating gene expression (Chighizola et al., 2019).

In summary, there are many different signal transduction pathways that are closely linked during mechanical transduction. Different mechanical signals may influence cellular responses in different ways through interconnected and mutually regulated signaling pathways. Mechanical stimuli depend on the above related biological basis and trigger some signaling pathways; however, the detailed mechanism of nerve cell response to mechanical stimulus needs to be further studied.

## Regulation of Physical Cues on Neuronal Behavior

Regulation of neuronal behavior by physical cues of remolded matrix are considered the most effective strategies to replace dead nerve cells and rebuild neural circuits for the repair of nerve injury. Currently, physical properties of the matrix, such as stiffness, mechanical tension, 2D terrain, and electrical conductivity, have been found to have positive effects on neuronal neurite growth and NSC differentiation. Here, we summarize the effect of various physical cues on the regulation of neural behavior.

### Stiffness

Stiffness refers to the ability of a material or structure to resist elastic deformation under stress. Many researchers have proposed a correlation between matrix stiffness and cellular behaviors after injury, disease, and cancer (Wu et al., 2019; Kayal et al., 2020; Prager et al., 2020). Further studies confirmed that the stiffness of the cellular environment affects cell adhesion, proliferation, migration, differentiation, and phenotype. Furthermore, matrix stiffness has a certain effect on neurite outgrowth (Wells, 2008; Khoshakhlagh and Moore, 2015). In particular, two general relationships have been proposed between the behavior of neurites and the stiffness of the matrix: (1) the growth of neurites is inversely proportional to the stiffness of the 2D substrate and the 3D hydrogel and (2) the maximal rate of neurite outgrowth is reached at an intermediate range of stiffness (Kim et al., 2015; Lantoine et al., 2016; Wen et al., 2018). In addition, neurites have a threshold response to matrix stiffness. Leach et al. (2007) cultured pheochromocytoma (PC12) cells on substrates with different stiffness to investigate the response of neurites to matrix stiffness. On the softest matrix (shear modulus ∼10 Pa), there were relatively few, short in length and unbranched neurites. On a harder matrix (shear modulus ∼10^2^–10^4^ Pa), neurites were longer and more branched, and the proportion of cells expressing neurites was higher. However, no significant difference was found in these measures on a substrate with a shear modulus of >10^2^ Pa (Leach et al., 2007).

**FIGURE 3:**
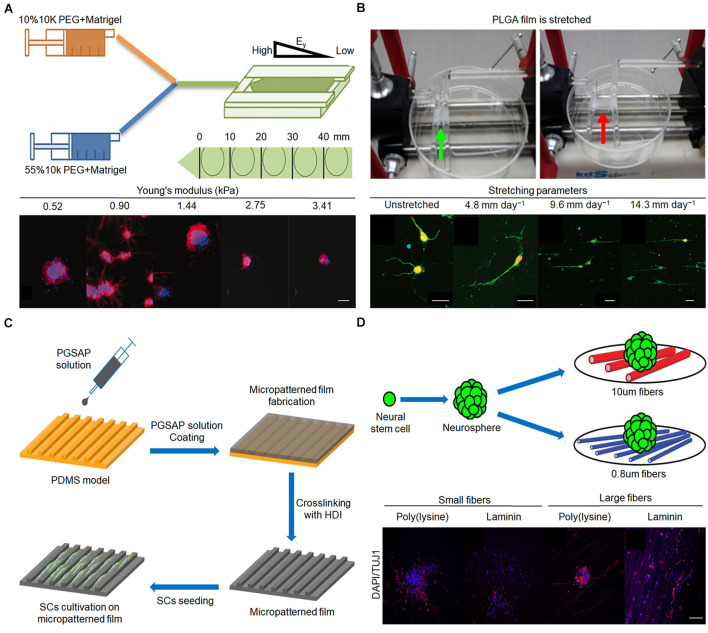
Effect of stiffness, mechanical tension, and 2D terrain on behaviors of nerve cells. **(A)** Fabrication procedure of gradient hydrogel. Immunofluorescent staining of tubulin III (red) and nucleus (blue) in human neural stem cell (hNSC) after 14 days for culture on PEG hydrogel possessing a continuous gradient in Young’s modulus (Mosley et al., 2017). Scale bar = 100 μm. **(B)** Poly(lactic-*co*-glycolic acid) (PLGA) film is stretched by the control system. Immunofluorescence staining of neurons after 3 days *in vitro* on upstretched film and films stretched at 4.8, 9.6, and 14.3 mm day^–1^ (Feng et al., 2016). Scale bar = 50 μm in all images. **(C)** Micropatterned film preparation and Schwann cell cultivation (Wu et al., 2018). **(D)** Mouse E13 NSCs were plated onto poly(ε-caprolactone) (PCL) fiber mat. Immunofluorescent staining of nerve cells on different diameters (Czeisler et al., 2016). Scale bar = 100 μm. Figures reproduced with permission from the references listed.

The stiffness of the matrix mainly affects the differentiation of NSCs into different cell phenotypes. When the stiffness of the matrix is approximately 1 kPa, NSCs are more easily differentiated into neurons (Rammensee et al., 2017; Blaschke et al., 2019). Leipzig and Shoichet (2009) developed a photopolymerized methylacrylamide chitosan biomaterial with a Young’s modulus adjustable from less than 1 kPa to greater than 30 kPa. The matrix was then co-cultured with neural progenitor cells, which proliferated the most on the surface with a Young’s modulus of 3.5 kPa. In the 1-kPa matrix, there were more mature neurons, and oligodendrocyte maturation and myelination were the best (Leipzig and Shoichet, 2009). Experiments by Blaschke et al. (2019) confirmed this view. They prepared polydimethylsiloxane (PDMS)-coated cell culture plates to simulate the physiological microenvironment of the living brain and produce varying degrees of elasticity, ranging from 1 to 50 kPa. Compared with the 50-kPa PMDS matrix, the proliferation rate of NSCs on 1 kPa PDMS was reduced by 45%. On 1 kPa PDMS, the number of neurons was 42%, while on 50 kPa PDMS, the number of neurons was 25%. Neurons from NSCs on 1 kPa PDMS showed 29% longer neurites compared with those on stiffer PDMS substrates, suggesting optimized neuronal maturation and an accelerated generation of neuronal networks (Blaschke et al., 2019). If the stiffness of the matrix exceeds or decreases below a certain range (∼1 kPa), the proportion of neurons will decrease. Mosley et al. (2017) found that the expression of tubulin III decreased to varying degrees (approximately 65% and 25%, respectively) when the matrix stiffness increased from 1.44 to 75 kPa or decreased from 1.44 to 520 kPa (Figure 3A). Furthermore, the response of NSCs to the stiffness of the matrix is not particularly sensitive and requires a larger span to be apparent. When the stiffness of the matrix was 15 and 50 kPa, the proportion of neurons was equal (Saha et al., 2008; Blaschke et al., 2019). In terms of NSC differentiation into glial cells, in general, the differentiation ability increased as the stiffness of the matrix increased. When the stiffness of the matrix increased from 1 kPa to 1 GPa, the proportion of astrocytes increased from 50 to 82% (Blaschke et al., 2019).

Currently, many different forms of matrices (e.g., electrospinning films, 3D printing scaffolds, and hydrogels) with various stiffnesses are implanted in the body for nerve repair. In particular, hydrogels are widely used because their stiffness can be precisely controlled by convenient methods such as adjusting the concentration of the solute or the illumination time. Fan et al. (2018) encapsulated induced pluripotent stem cell-derived NSCs (iNSCs) in light crosslinking poly(ethylene glycol) (PEG)–methacrylate gelatin (GelMA) with different stiffnesses. iNSCs encapsulated in 3D hydrogels survived and differentiated well *in vitro*. In low-modulus hydrogels, neurite growth was more vigorous and neuronal differentiation was more obvious. Furthermore, the GelMA/iNSC hydrogel was implanted into the mouse spinal cord transection model and was found to have a significant therapeutic effect on promoting axon regeneration while inhibiting the formation of glial fibrillary acidic protein (GFAP)-positive cells and glial scar and significantly promoting functional recovery (Fan et al., 2018).

### Mechanical Tension

Mechanical tension is the mutual traction force existing on the contact surface between two adjacent parts of the inside of an object when the object is under tension (Ahuja et al., 2017a). The idea that mechanical tension is involved in nervous system morphogenesis was first revealed in the late 1970s, and traction neurites were used in the first attempts to apply mechanical stimulation to nerve cells. Studies have shown that force is involved in growth cone-mediated axon growth and guidance, as well as stretch-induced elongation when an organism increases in size after forming an initial synaptic connection (Athamneh and Suter, 2015). Currently, mechanical tension is applied to nerve cells through various strategies. For instance, a micromanipulator can precisely control the mechanical force applied to specific single cells, allowing neurites to grow and extend quickly. However, the feasibility of the micromanipulator is limited because the setup of the micromanipulator is complicated, and it is difficult to maintain the stability of the force for long-term observation. In recent years, to achieve a better control effect, the application of mechanical tension has changed. The stimulus of mechanical tension is transmitted indirectly to the cell through application to the matrix, rather than directly acting on a single nerve cell. Some researchers have studied nerve cell mechanics by stretching elastic membranes. This flexible membrane installed in the translation stage can transmit uniform stress and stable strain to cells in long-term experiments, and the reliable setup of the platform overcomes the inconvenience of the micromanipulator (Athamneh and Suter, 2015). Furthermore, the mechanical tension exerted on nerve cells can be indirectly assessed by more controllable factors, such as the amplitude of the matrix shape change (Arulmoli et al., 2015).

During stretching, the elongation of neurites is obviously enhanced, the number of neurites is reduced, and the growth direction of neurites tends to be parallel. Feng et al. (2016) cultured E15 spinal motor neurons on poly(lactide-*co*-glycolide) membranes with stretched parameters of 4.8, 9.6, and 14.3 mm day^–1^. The neurite grew longer and reached 209 μm, an increase of 113% in length, when the mechanical tension was 14.3 mm day^–1^ compared with the unstretched group (Figure 3B). Furthermore, the primary motor neurons on unstretched matrix had an average of 4.4 neurites, whereas only 2.5, 2.2, and 2.7 neurites were found when the matrix was stretched with 4.8, 9.6, and 14.3 mm day^–1^ as parameters, respectively (Feng et al., 2016). The parallelism between the direction of neurite growth and the direction of mechanical tension increases to a certain extent when the mechanical tension gradually increases from 4.8 mm day^–1^ (Chang et al., 2013; Feng et al., 2016). In addition, it is worth noting that the diameter of the axon does not decrease after application of mechanical tension (Anava et al., 2009). In addition to guiding neurites, mechanical tension can promote the elongation of NSCs and enhance the differentiation of NSCs into mature neurons. When mechanical tension that can only cause static deformation of nerve cells is applied, the differentiation of NSCs incubated on a specific ECM into oligodendrocytes is inhibited; however, the differentiation into neurons and astrocytes is not affected (Chang et al., 2013). Mechanical tension causes nerve cells to undergo dynamic deformation, which is beneficial for the directional differentiation of NSCs into neurons (Arulmoli et al., 2015). Since few studies have examined the effect of mechanical tension on the differentiation of NSCs, the conclusions need to be further evaluated.

### Two-Dimensional Terrain

The 2D terrain is a general term for various micropatterns of the matrix surface (Zhang et al., 2018). Nerve cells in contact with the surface micropatterns of the matrix are subjected to pressure that is asymmetric and irregular compared to the flat surface, thus initiating the process of converting mechanical stimuli into biochemical signals that influence nerve cell behavior (Jeon et al., 2014; Kim et al., 2018). Advances in manufacturing technology have resulted in a number of 2D terrains designed to guide the behavior of nerve cells, such as nerve templates, honeycombs, oriented fiber arrangements, and grooves. In particular, oriented fibers and grooves are widely used owing to their mature fabrication technology (Tsuruma et al., 2008; Du et al., 2014; Yang et al., 2014). For instance, electrospinning technology can easily and quickly prepare oriented nanofiber scaffolds with highly consistent alignment directions (Panahi-Joo et al., 2016). Photolithography is also widely used to prepare micropatterned surfaces because of the advantages of simplified operation and accurate control of the ditches, ridges, and heights of grooves during the preparation process (Yang K. et al., 2015).

The 2D terrain regulates the growth of neurites mainly through physical contact to guide the growth direction of neurites (Wang et al., 2015; Yao et al., 2016). Neurites grow randomly on unaligned fibers and highly parallel on aligned fibers (Lins et al., 2017). Zhang et al. (2021) prepared conductive composite fibers composed of poly(ε-caprolactone) (PCL) and carbon nanotubes (CNTs) with high orientation by electrospinning at different rotational speeds (500, 1,000, and 2,000 rpm). In particular, the overall morphology of PC-12 neural cells seeded on PCL/CNT–composite fibers at 1,000 rpm was visualized by microscopy after 7 days, revealing a spindle-spreading morphology as well as an extension of the filamentous cytoskeleton. Additionally, the direction of cell elongation was precisely parallel to the orientation direction of the fibers, indicating that a substrate architecture with longitudinally aligned topography may support spatial cell proliferation *in vitro* through contact guidance (Zhang et al., 2021). Grooves can guide the growth direction of neurites through the walls of the grooves to limit the growth of neurites, but grooves with different shapes have different directional guiding effects on neurites. Mobasseri et al. (2015) designed various surface topographies, including sloped (SL), V-shaped, and square-shaped wall grooves, using PCL and polylactic acid (PLA) blended films. Compared with the square-shaped grooves, the V-shaped and sloped wall grooves led to better effects on the orientation of neurite growth (Mobasseri et al., 2015). In addition, neurons can grow in different orientations even in grooves of the same shape, due to differences in the ditches, ridges, and heights. When the width of the ditch and nerve cell is similar, and the ridge is narrow, the nerve cell can only grow inside the ditch and exhibit the best orientation of neurite growth. If the ditch is much wider than the width of a single cell, nerve cells can grow randomly in the ditch and show a slight effect on the orientation of neurite growth. Compared with poly(glycerol sebacate)-*co*-aniline pentamer (PGSAP)-flat films, Schwann cells cultured on micropatterned PGSAP films exhibited better alignment behavior. In particular, as high as 90% and 73% of the cells were within ±10° orientation on PGSAP-50 (groove/ridge: 50/50 μm) and PGSAP-100 (groove/ridge: 100/50 μm) films, respectively (Figure 3C; Wu et al., 2018).

In contrast to random fibers, well-arranged fibers provide a topographic cue to stimulate elongation and enhance neuronal differentiation of adult NSCs and mesenchymal stem cells (Yao et al., 2016). In addition, fibers with larger diameters modified by specific substances are more conducive to the differentiation of NSCs into neurons while inhibiting their differentiation into glial cells. As shown in Figure 3D, NSCs were co-cultured with fibers of different sizes modified with poly(D-lysine) and laminin, the average goodness of fit of β-tubulin III gene expression in cells on 10 μm fiber modified by laminin was greater than 0.8 μm, and the average goodness of fit of GFAP gene expression in cells on 10 μm fiber modified by poly(D-lysine) was less than 0.8 μm. The proportion of neurons increased by approximately 20% and the proportion of glial cells decreased by approximately 10% on the 749-nm fiber compared with that on the 283-nm fiber (Czeisler et al., 2016). Groove or porous structures alone have little effect on NSC differentiation. However, the combination of the groove and porous structure can play a synergistic role in promoting the differentiation of NSCs into neurons (Tsuruma et al., 2008; Yang et al., 2014). In addition, the specific protein levels of neurons in composite structural matrices are approximately 80–100% higher than those on single microgrooves or nanopore matrices (Yang et al., 2014).

In addition to verifying the regulation of nerve cells by the 2D terrain at the cellular level, the linearly oriented 2D terrain is widely integrated into the scaffold to repair nerve injury. Koffler et al. (2019) used a microscale continuous projection printing method to prepare a complex central nervous system structure scaffold that can have precise and highly linearly arranged linear channels to simulate the linear organization of white matter. Furthermore, the scaffold was loaded with neural progenitor cells and implanted into rodents, where highly linear axons were subsequently found. In addition, the axon of the injured host was regenerated into the 3D bionic scaffold, and the neural progenitor cells in the implanted scaffold extended the axon from the scaffold to the host spinal cord below the injury, forming synaptic transmission and significantly improving function (Koffler et al., 2019).

### Electrical Conductivity

Nerve tissues are in the microelectric microenvironment, which is critical in maintaining normal physiological functions (Stewart et al., 2015). The growth, differentiation, and migration activities of embryonic and adult nerve cells mainly depend on electrical conductivity, synapse formation, and the survival of neurons during the last stages of development. Electrical conductivity is related to ion channels in the plasma membrane that are responsible for triggering the signaling pathways between neurons, and it seems to play important roles in various stages of neural development. In particular, the physiological function of the mammalian central nervous system is highly dependent on the electroresponsive properties of single neurons. In some of these cells, the ionic conductances responsible for their excitability also endow them with autorhythmic electrical oscillatory properties. In addition, chemical or electrical synaptic contacts between these neurons often result in network oscillations. The conductive matrix with ionic conductances can be used to promote the exchange of ions inside and outside the cells, the receptors on the cell surface being rearranged, and the deposition of active molecules and proteins on the membrane (Yu et al., 2008). Activation *via* phosphorylation of ERK1/2 and increasing the expression of early growth response protein 1 gene by NGF is one of the possible mechanisms for neuronal growth through electrical stimulation. It was previously claimed that activating PKC by electrical stimulation can also control neurite outgrowth. PKC through NGF-induced pERK1/2 pathway enhances neurite outgrowth (Farokhi et al., 2020). By using electrical stimulation, it is also possible to induce the regeneration of axons after axotomy by enhancing the expression of brain-derived neurotrophic factor. This phenomenon occurs by activation of Ca^2+^ and Erk-dependent signaling pathways. The high amounts of Ca^2+^ in intracellular elevate the level of intracellular cAMP. The high level of cAMP and Ca^2+^ provokes the regeneration of axon by mediating the fusion of axonal fragments and stimulating the postsynaptic branching. The cAMP pathway is linked to many other signaling pathways including phosphatase and tensin homolog, MAPK, and signal transducer and activator of transcription 3 that concurrently induce neurite outgrowth and promote axon regeneration (Brandl et al., 2007; Pires et al., 2015).

Conductive biomaterials can not only simulate the microelectric microenvironment of the body and maintain the integrity of the electrical pathway, but can also combine with external electrical stimulation to synergistically regulate the physiological activities of nerve cells, affecting proliferation, migration, differentiation, and neurite outgrowth, which provides a new direction for nervous tissue repair (Arioz et al., 2018; Zhu et al., 2018). In particular, conducting polymers have been used in biomedical research since the 1990s because of their advantages such as easy preparation, stable electrical properties, good biocompatibility, and controllable release of biomolecules. In the last 10 years, the trend in the development of conductive polymers has reflected the strong research interest and enthusiasm of the researchers and proves the importance of conducting polymers as biomaterials and their application potential in tissue repair. Polypyrrole (PPy), polyaniline (PANI), and poly(3,4-ethylenedioxythiophene) (PEDOT) have been the most widely used conductive polymers in recent years (Xu et al., 2016; Wang et al., 2017; Lee et al., 2018; Chen et al., 2019). These conductive materials are often used in conjunction with other polymers [e.g., PLA, PEG, chitosan (Cs), and polystyrene] to prepare a conductivity matrix (e.g., films, scaffolds, and hydrogels) by electrospinning, 3D printing, or microemulsion polymerization methods (Gajendiran et al., 2017; Gupta et al., 2019). For instance, Sudwilai et al. (2014) used a mixture of PPy and PLA to prepare conductive scaffolds by electrospinning. In addition, the conductivity of the conductive matrix can be adjusted by controlling the doping of the oxidant, the adsorption and dispersion of the conductive substance, and so forth (Nguyen et al., 2014; Zhou L. et al., 2018).

According to the reported results, the electrical conductivity that affects the number and length of neurites is mainly concentrated in the range of 10^–10^–10^–3^ S cm^–1^ (Zhou Z. et al., 2018; Chen et al., 2019). With the upregulation of electrical conductivity, the length and number of neurites increase disproportionately. Arioz et al. (2018) developed a supramolecular electroactive nanosystem containing tetra aniline peptide nanofibers, which exhibited electroactivity with conductivity values of 6.97 × 10^–6^ S cm^–1^. Compared with the matrix with a conductivity of 1.10 × 10^–10^ S cm^–1^, the neurite length of PC12 cells co-cultured with the supramolecular electroactive nanosystem could be more than doubled, and the percentage of neurite-bearing PC12 cells was increased by 10%. However, the growth of the neurite does not increase with a further increase in electrical conductivity (Arioz et al., 2018). In addition, the conductive matrix combined with exogenous electrical stimulation further improved the effect of neurite outgrowth. PC12 cells were cultured on the biomimetic PEDOT matrix; when the applied voltage was 20, 40, and 60 mV, neurite outgrowth experienced substantial increases, and when the applied voltage was 60 mV, the median neurite length reached a maximum of 35.7 μm, corresponding to a 124% enhancement of the original value (15.9 mm) on PEDOT (Zhu et al., 2014).

**FIGURE 4:**
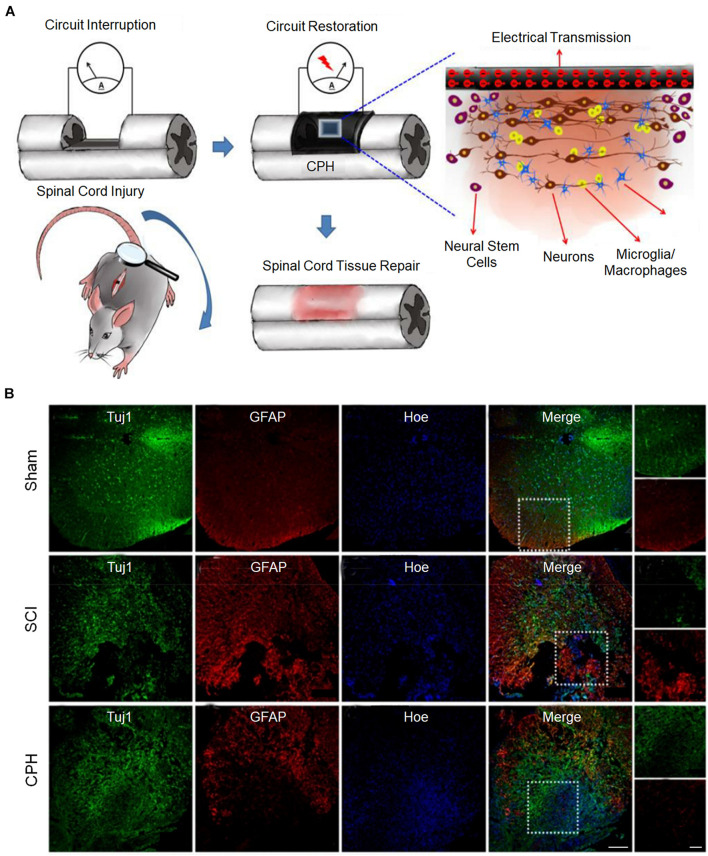
Conductive hydrogels repair spinal cord injury (Zhou L. et al., 2018). **(A)** The possible mechanism for conductive hydrogel to promote tissue regeneration is to restore the interrupted spinal circuit through endogenous neurogenesis. **(B)** Immunohistofluorescence images of transverse spinal cord sections obtained from animals in the sham, injury, and hydrogel groups at 6 weeks. Scale bars = 200 and 100 μm, respectively. Figures reproduced with permission from the references listed.

The conductivity of the matrix mainly regulates the differentiation direction of NSCs. In general, when the conductivity of the matrix increases, the ability of NSCs to differentiate into neurons increases, but the enhancement may be tortuous (Zhou Z. et al., 2018). Based on plant-derived polyphenols and PPy, a highly conductive (0.05–0.18 S cm^–1^) polymer hydrogel was developed, which can accelerate the differentiation of NSCs into neurons and inhibit the development of astrocytes *in vitro* (Figure 4A). The proportion of neurons was the highest when the conductivity was 0.18 S cm^–1^. However, when the conductivity was 0.12 S cm^–1^, the proportion of neurons was lower than that when the conductivity was 0.05 S cm^–1^. After implantation of the conductive polymer hydrogels into the rats of the spinal cord injury hemisection model, it was found that conductive polymer hydrogels can also activate the endogenous neurogenesis of NSCs in damaged areas (Figure 4B) and significantly restore their motor functions (Zhou L. et al., 2018). In addition, in a matrix with electrical conductivity, while promoting differentiation of NSCs into neurons, the differentiation into glial cells is inhibited (Wang et al., 2017; Zhou L. et al., 2018). When NSCs were co-cultured with PEDOT/Cs/gel and Cs/gel scaffolds, the relative gene expression levels of β-tubulin III and GFAP in cells with the PEDOT/Cs/gel scaffolds were approximately 2.76 and 1.96 times higher, respectively, than those in cells with the Cs/gel scaffolds (Wang S. et al., 2018).

## Concluding Remarks and Future Perspectives

The physical cues provided by biomaterial matrix have a significant effect on the behavior regulation of nerve cells and show considerable advantages. First, physical cues are quite economical and effective in the process of preparation and use. Second, in addition to a single physical cue, multiple physical cues can play a synergistic effect. Third, different physical cues have different regulatory focuses, and the biomaterial matrix can be personalized and tailored according to requirements. Suitable physical cues can not only provide a livable microenvironment for transplanted or autologous nerve cells but also synergistically improve the therapeutic effect with biochemical cues.

However, research on the regulation of neural cells by various physical cues is scarce and fragmented currently. We have a shallow understanding of the role played by different physical cues in the regulation process, and no definite consensus has been reached. The key questions remain unanswered, such as what is the most suitable conductivity for conductive materials, what happens to the most suitable electrical stimulation when conductivity changes, and whether the optimal stiffness of different materials is the same despite the lack of a comparative test. These are not conducive to the precise regulation of nerve cells, which may become uncontrollable factors in neural repair. In addition, NSCs differentiate into a large proportion of neurons and the longest neurite, which we are currently pursuing. However, during nerve repair, we should consider various factors to seek the best state after balancing in all aspects to achieve the best nerve repair effect. These questions are the directions we should strive to explore in the future.

Although we are still in the early stages of revealing the effects of physical cues on nerve regeneration, with the deepening of our understanding of nerve injury and the advancement of manufacturing technology, it is very promising to achieve excellent nerve repair in clinical practice.

## Author Contributions

YL wrote the initial manuscript. CF and YL contributed new ideas. YX and HW created the figures. YL and JL created Table 1. YL, JL, and BL revised the manuscript and approved the final version. All authors read and approved the final manuscript.

## Conflict of Interest

The authors declare that the research was conducted in the absence of any commercial or financial relationships that could be construed as a potential conflict of interest.

## Publisher’s Note

All claims expressed in this article are solely those of the authors and do not necessarily represent those of their affiliated organizations, or those of the publisher, the editors and the reviewers. Any product that may be evaluated in this article, or claim that may be made by its manufacturer, is not guaranteed or endorsed by the publisher.
